# Analysis of Parkinson disease patients from Portugal for mutations in SNCA, PRKN, PINK1 and LRRK2

**DOI:** 10.1186/1471-2377-8-1

**Published:** 2008-01-22

**Authors:** Jose Bras, Rita Guerreiro, Maria Ribeiro, Ana Morgadinho, Cristina Januario, Margarida Dias, Ana Calado, Cristina Semedo, Catarina Oliveira, John Hardy, Andrew Singleton

**Affiliations:** 1 Center for Neurosciences and Cell Biology, Faculty of Medicine, University of Coimbra, Coimbra, Portugal; 2 Neurology Service, Coimbra University Hospital, Coimbra, Portugal; 3 Laboratory of Neurogenetics, National Institutes on Aging, National Institutes of Health, Bethesda, Maryland, USA; 4 Neurology Service, Lisbon Hospital Center – Center Region EPE, Lisbon, Portugal

## Abstract

**Background:**

Mutations in the genes *PRKN *and *LRRK2 *are the most frequent known genetic lesions among Parkinson's disease patients. We have previously reported that in the Portuguese population the *LRRK2 *c.6055G > A; p.G2019S mutation has one of the highest frequencies in Europe.

**Methods:**

Here, we follow up on those results, screening not only *LRRK2*, but also *PRKN, SNCA *and *PINK1 *in a cohort of early-onset and late-onset familial Portuguese Parkinson disease patients. This series comprises 66 patients selected from a consecutive series of 132 patients. This selection was made in order to include only early onset patients (age at onset below 50 years) or late-onset patients with a positive family history (at least one affected relative). All genes were sequenced bi-directionally, and, additionally, *SNCA*, *PRKN *and *PINK1 *were subjected to gene dosage analysis.

**Results:**

We found mutations both in *LRRK2 *and *PRKN*, while the remaining genes yielded no mutations. Seven of the studied patients showed pathogenic mutations, in homozygosity or compound heterozygosity for *PRKN*, and heterozygosity for *LRRK2*.

**Conclusion:**

Mutations are common in Portuguese patients with Parkinson's disease, and these results clearly have implications not only for the genetic diagnosis, but also for the genetic counseling of these patients.

## Background

Parkinson's disease (PD) is clinically characterized by resting tremor, rigidity, bradykinesia, and postural instability [1]. The gold standard of PD diagnosis remains a pathological one. Pathological hallmarks include cell loss in pigmented nuclei and the formation of eosinophilic intra-cytoplasmic inclusions, termed Lewy bodies, particularly in the dopaminergic neurons of the substantia nigra pars compacta [2,3]. So far, mutations in *SNCA *(Alpha-synuclein; OMIM#163890), *PRKN *(Parkin OMIM#602544), *DJ1 *(Oncogene DJ1 OMIM#602533), *PINK1 *(PTEN-Induced Putative Kinase 1 OMIM#608309) and *LRRK2 *(Leucine-Rich Repeat Kinase 2 OMIM#609007) have been implicated as causes of monogenic PD [4-9].

A missense mutation within *SNCA *encoding the protein alpha-synuclein was the first identified genetic cause of PD [6] and copy number mutations at this locus have also been shown to cause familial PD [10]. Mutations in *PRKN *are a common cause of autosomal recessive early-onset parkinsonism [11,7], however mutations in *PINK1 and DJ-1 *have also been reported as associated with this type of parkinsonism, although only five causative mutations have been described in the latter [12-14]. Mutations in the gene *LRRK2 *are a common cause of autosomal dominant and apparently sporadic PD. The *LRRK2 *mutation c.6055G > A; p.G2019S, accounts for 1–2% of typical sporadic PD in the North American and Northern European white population, with higher prevalence in Portuguese (6%), Ashkenazi Jewish (18.3%) and North African Arab populations (39%) [15-18].

We had previously shown that the c.6055G > A; p.G2019S mutation in *LRRK2 *is common in Portuguese PD patients [15]. Here we extend upon this study to search for additional *LRRK2*, *SNCA*, *PRKN *and *PINK1 *mutations in a clinic-based cohort of patients, mainly from the central region of Portugal.

## Methods

After obtaining informed consent, 132 PD patients underwent a standardized neurological examination by a movement disorder specialist. The diagnosis of PD was based on the UK Brain Bank diagnostic criteria (family history was not used as an exclusion criterion) and those published by Gelb et. al. [2,19]. Family history was considered positive if parkinsonism was reported in at least a first- or second-degree relative. Collection of these 132 patients was performed at the Movement Disorder Clinics of both the University of Coimbra Hospital and the Lisbon Hospital Center – Center Region EPE in Lisbon, in a consecutive manner, all patients consent to participate. This cohort is identical to that previously described by us except for the inclusion of 4 additional PD patients [15]. From this series of 132 subjects we have selected 66 unrelated patients to include only those with a positive family history for parkinsonism, or early-onset disease (age at onset <50 years of age). The remaining 66 patients failed to meet either of these criteria, were related to a proband already included or had previously been found to carry the *LRRK2 *c.6055G > A; p.G2019S mutation (n = 11). This selection led to the inclusion of 39 patients with positive family history and 46 patients with early-onset PD; 19 patients presented with both an early-onset phenotype and a positive family history, thus the net number of patients from both inclusion groups is 66 (Table 1).

**Table 1:** Features of patients studied, where more than one affected member was identified in a family only the presenting affected member (proband) is included.

Characteristic	Subjects (n = 66)
Age at collection (mean ± SD)	60.1 ± 11.1
Age at onset (mean ± SD)	44.5 ± 9.3
Range of age at onset	20 – 60
Family history	
Positive	39 (59.1)
Negative	27 (40.9)

Additionally we have included a control group comprised of 126 healthy subjects as previously described [15]. Briefly, this control group consisted primarily of spouses accompanying patients to the clinic (~80%); the remaining controls were recruited from non-neurology outpatient clinics, after observation by the movement disorders specialist. This series presented a mean age of 60.5 ± 23.1 years. Apart from the spouses of the patients, no other familiarity with movement disorders patients was found. All individuals are Caucasian and of apparent Portuguese ancestry.

Genomic DNA was extracted from peripheral blood using standard methods. We screened the genes *SNCA*, *PRKN*, *PINK1 *and *LRRK2 *for sequence variants and, with the exception of *LRRK2*, for genomic copy number variants. The reference sequence used for the *PRKN *gene throughout this paper is based on the accession number NM_004562 and codon counting starts from the first ATG.

For *SNCA*, *PRKN *and *PINK1*, all exons were polymerase chain reaction-amplified and sequenced in both directions using BigDye chemistry (Applied Biosystems, Foster City, CA) on an ABI 3100 Genetic Analyzer as previously described [7,20,21]. While for the *LRRK2 *gene, only exon 41 was screened for mutations, using conditions previously described [15].

Gene dosage analysis was performed using the ABI 7900 Sequence Detection System. Exons 1,2, 4–9 and 11–12 of *PRKN *and exons 1 and 2 of *SNCA*, as well as the complete coding region of *PINK1 *were individually co-amplified with β-globin, which served as an endogenous reference gene. Each plate contained six replicates of every genomic DNA sample, control DNA, and a no-template water control. The cycle in the log phase of PCR amplification at which a significant fluorescence threshold was reached (*Ct*) was used to quantify each exon relative to β-globin. The dosage of each exon relative to β-globin and normalized to control DNA was determined using the 2^-ΔΔ*Ct *^method (Applied Biosystems, Foster City, CA).

## Results

**Figure 1:**
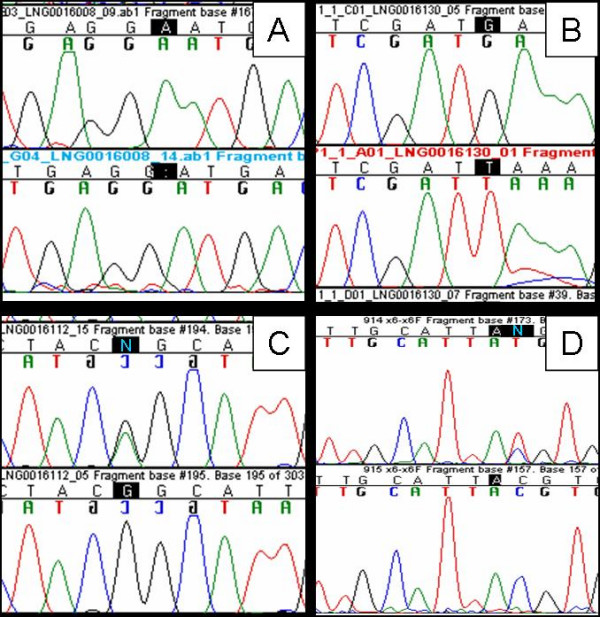
**Electropherograms of the pathogenic point mutations found**. image of the four pathogenic variants discovered in our series. A) chromatogram of the homozygous 154delA in *PRKN *exon 2, predicted to cause the N52fsX80 aminoacid change; B) chromatogram of the new mutation G1183T in *PRKN *exon 11, apparently homozygous, predicted to cause a premature stop codon at position 395; C) chromatogram of the heterozygous G6055A in *LRRK2 *exon 41, predicted to cause the G2019S change; D) chromatogram of the heterozygous C719T variant in *PRKN *exon 6, predicted to cause the T240M change.

Analysis of both sequence and copy number yielded several parkin mutations in our subset of patients. The positive results found are represented in Table 2, and the electropherograms corresponding to point mutations are shown in Figure 1. We found four patients in whom both alleles were mutated; three of these patients had the same homozygous mutation (c.154delA; p.N52fsX80), a single base pair deletion that inserts a premature stop codon downstream; one patient showed deletion of exon 2 and duplication of exon 5; analysis of a sixth sample (S4) showed data consistent with a homozygous c.1183G > T; p.E395X mutation and a duplication of exon 9; because the co-occurrence of 3 mutations in the same gene is unlikely we designed an additional forward primer that flanked the E395X mutation as close as possible on the 5' side; sequencing of the PCR product generated by amplification with this new primer and primer PRKNexon11R showed the E395X in a heterozygote state, suggesting that the duplication of exon 9 (and presumably exon 10, which we were unable to assay) interfered with the original PCR and sequencing reaction. Thus in Table 2 this mutation is denoted as a compound heterozygous E395X/exon 9 duplication mutation.

**Table 2:** Variants found

Sample	Gene	Nucleotide change	Amino acid change	Copy variation	Exon	Zygosity	AAO
S1	*PRKN*	154delA	N52fsX80	N/A	2	Homo	30
S2	*PRKN*	154delA	N52fsX80	N/A	2	Homo	35
S3	*PRKN*	154delA	N52fsX80	N/A	2	Homo	21
S4	*PRKN*	G1183T	E395X*	N/A	11	Het	53
	*PRKN*	N/A	N/A	Duplication	9	Het	
S5	*PRKN*	C719T^#^	T240M	N/A	6	Het	55
S6	*PRKN*	N/A^#^	N/A	Duplication	8	Het	33
S7	*PRKN*	N/A	N/A	Duplication	9	Het	38
	*LRRK2*	G6055A	G2019S	N/A	41	Het	
S8	*PRKN*	N/A^#^	N/A	Deletion	8 – 11	Het	32
S10	*PRKN*	N/A^#^	N/A	Deletion	2	Het	35
	*PRKN*	N/A^#^	N/A	Duplication	5	Het	
S12	*LRRK2*	G6055A	G2019S	N/A	41	Het	41

Homo: Homozygosity; Het: heterozygosity; * previously undescribed mutation; ^# ^Variants of unknown significance e.g pathogenicity not confirmed herein. AAO: Age at onset.

Two samples tested positive for the *LRRK2 *c.6055G > A; p.G2019S mutation, sample S12 and sample S7. Notably, analysis of sample S7 also showed a heterozygous duplication of *PRKN *exon 9. Screening of variants E395X, G2019S and 154delA in 252 control chromosomes failed to reveal any control subjects harboring these mutations.

Additionally, we have identified three patients with heterozygous mutations in *PRKN*: one harboring the T240M variant; another with a deletion of exons 8 through 11 and one with an exon 8 duplication. Furthermore, we have found one patient with two heterozygous dosage variants: an exon 2 deletion and an exon 5 duplication.

No mutations were found in *SNCA *or *PINK1*.

## Discussion

We present in this study a detailed mutation analysis of *PRKN*, *PINK1, SNCA *and *LRRK2*. We have included PD patients with a positive family history (n = 19 age at onset <50 years, n = 20 age at onset ≥ 50 years), or early-onset sporadic disease (n = 27) in order to maximize our chances of identifying mutations. This approach has led us to find 6 subjects (9.1%) with pathogenic mutations in *LRRK2 *or *PRKN*, in addition to 4 variants of unknown significance in 4 patients.

In our previous report [15] we showed that the c.6055G > A; p.G2019S LRRK2 mutation underlies about 6% of late-onset PD in the Portuguese population. While we did not find any c.6055G > A; p.G2019S carriers in the 20 late-onset patients studied here, we did identify c.6055G > A; p.G2019S in 2 of 46 early-onset cases. One of these individuals also carried a heterozygous duplication of *PRKN *exon 9 consistent with the notion of digenic parkinsonism, as previously described [22]. This patient presented no family history consistent with PD, while the other *LRRK2 *patient had positive family history. Taking into account the removal of samples previously found to carry the c.6055G > A; p.G2019S mutation we calculate that this mutation is present in 9 probands out of the entire series of 132 patients; this represents 2 of 46 early-onset patients, counting only sporadic cases and a single proband from each family (4.3%) and 7 of 76 late-onset patients counting only sporadic cases and a single proband from each family (9.2%).

**Figure 2:**
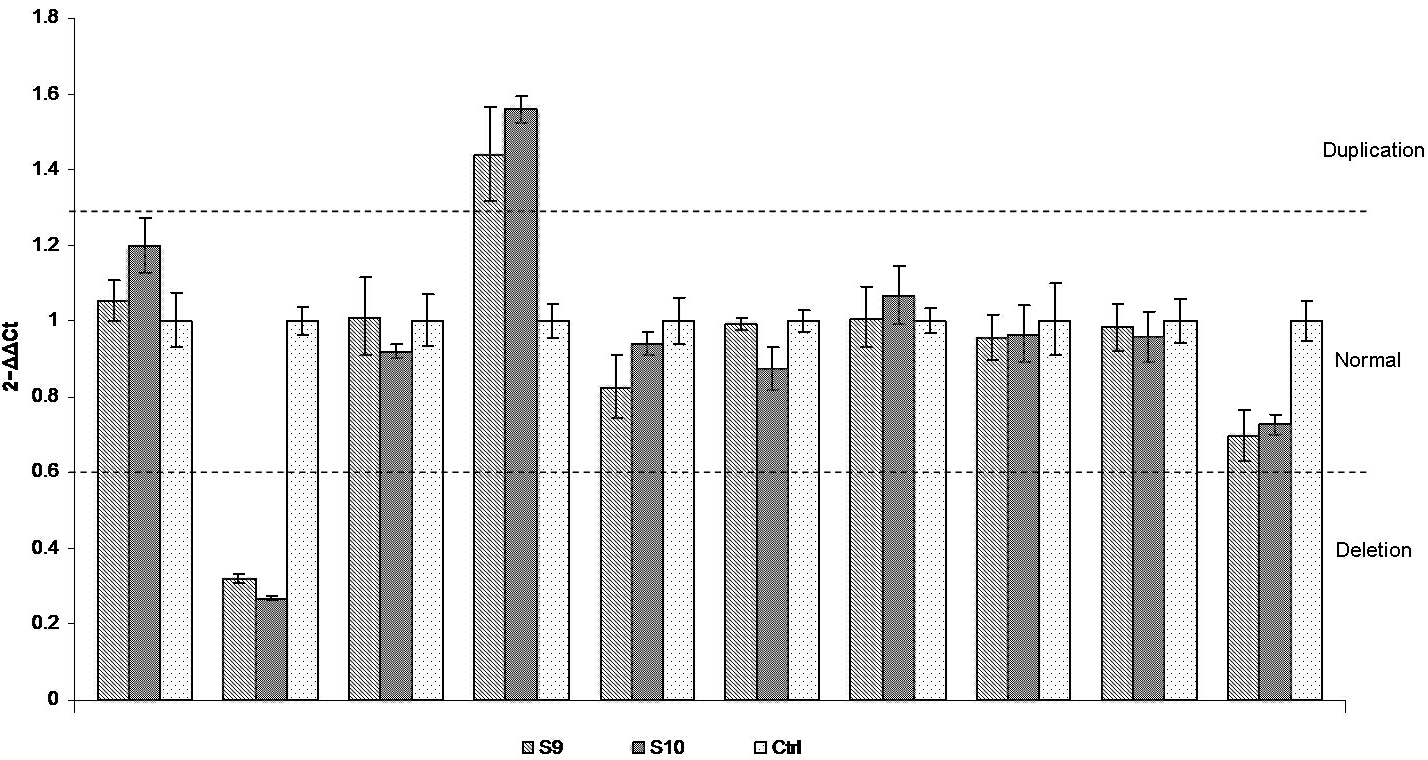
**Dosage plots**. Dosage plot of the variants found in PRKN in sample S10 and its sibling here denoted as S9. Represented are exons 1, 2, 4–9, 11 and 12. Each exon was compared with the beta-globin gene and normalized to a control DNA.

We found several *PRKN *mutations as either homozygous or compound heterozygous loss of function changes. The N52fsX80 variant was the most frequent mutation identified in *PRKN*. It was present as a homozygous mutation in three unrelated young onset patients (of 46, 6.5%). Analysis of relatives of these patients failed to show any heterozygous carriers of this mutation with parkinsonism. We identified a heterozygous deletion of exon 2 and a duplication of exon 5 in *PRKN *in one early-onset patient with positive family history. The only affected family member that was available for testing was the sibling of S10 who presented with the same two variants, albeit with a remarkably different age at onset (50 years vs 35 years of patient S10) (Figure 2). While parsimony suggests that these mutations are *in trans *we were unable to unequivocally establish phase as DNA was unavailable from other family members. We also identified a heterozygous deletion of exons 8 through 11 in a female patient with an age at onset of 32 years. Additional family members were unavailable, so we were unable to determine whether this mutation represented a single contiguous mutation or two mutations existing *in trans *and thus the pathogenicity of the observed changes remains unknown. We identified a novel mutation in *PRKN *exon 11 (E395X) as a heterozygous alteration. This patient also possessed a heterozygous duplication of exon 9. The pathogeneicity of the new E395X mutation is clear since it is a nonsense mutation that occurs upstream of a functional domain of the protein. Of these patients presenting either homozygous or clear compound heterozygous mutations in *PRKN*, four (80%) have an age at onset below 40 years. Only one (patient S4) presents late-onset disease (53 years).

In addition we identified several *PRKN *variants of unknown significance. The T240M alteration, an exon 8 deletion and an exon 8 duplication were each identified as heterozygous mutations in single patients. In the absence of additional mutations in *PRKN *in these subjects, we have not considered these as disease causing variants in these patients. We make this statement with caution, since we cannot rule out copy number mutations in exons 3 or 10, which we were unable to assay effectively.

## Conclusion

It is now clear that genetics plays an important role in the pathogenesis of PD. Specifically, in the Portuguese population, we have found a reasonable number of mutations: the frequency of the c.6055G > A; p.G2019S is one of the highest in Europe, and in the present paper we have found that 8.7% (4 out of 46 cases) of early-onset cases are attributable to *PRKN *mutations. Similar to other reports we found *PINK1 *and *SNCA *mutations to be a rare cause of disease in our families [23]. Taken as a whole these results have implications mainly for clinicians in Portugal; in particular showing that genetic screening may aid the diagnosis of PD in this population. However, even with the combination of gene dosage and sequencing, a significant proportion of mutations might remain undetected, probably due to the size and the complexity of the *PRKN *gene. In this way, negative results should be interpreted with caution, as well as heterozygous mutations in this gene.

## Competing interests

The author(s) declare that they have no competing interests.

## Authors' contributions

JMB and RJG performed the genotyping, and drafted the manuscript. MHR, CJ, AM, MD, AC and CS contributed to collecting materials. CRO, JH, and AS participated in the study design and coordination, together with drafting the manuscript. All authors read and approved the final manuscript.

## Pre-publication history

The pre-publication history for this paper can be accessed here:


